# Why Chloroplasts and Mitochondria Contain Genomes

**DOI:** 10.1002/cfg.245

**Published:** 2003-02

**Authors:** John F. Allen

**Affiliations:** Plant Biochemistry Center for Chemistry and Chemical Engineering Lund University Box 124 Lund SE-221 00 Sweden

## Abstract

Chloroplasts and mitochondria originated as bacterial symbionts. The larger, host cells acquired genetic information from their prokaryotic guests by lateral gene transfer. The prokaryotically-derived genes of the eukaryotic cell nucleus now function to encode the great majority of chloroplast and mitochondrial proteins, as well as many proteins of the nucleus and cytosol. Genes are copied and moved between cellular compartments with relative ease, and there is no established obstacle to successful import of any protein precursor from the cytosol. Yet chloroplasts and mitochondria have not abdicated all genes and gene expression to the nucleus and to cytosolic translation. What, then, do chloroplast- and mitochondrially-encoded proteins have in common that confers a selective advantage on the cytoplasmic location of their genes? The proposal advanced here is that co-location of chloroplast and mitochondrial genes with their gene products is required for rapid and direct regulatory coupling. Redox control of gene expression is suggested as the common feature of those chloroplast and mitochondrial proteins that are encoded *in situ*. Recent evidence is consistent with this hypothesis, and its underlying assumptions and predictions are described.


**Comparative and Functional Genomics**
*Comp Funct Genom* 2003; **4**: 31–36.
Published online in Wiley InterScience (www.interscience.wiley.com). **DOI:** 10.1002/cfg.245

**Conference Review**

### Why chloroplasts and mitochondria contain genomes

John F. Allen*
*Plant Biochemistry, Center for Chemistry and Chemical Engineering, Lund University, Box 124, SE-221 00 Lund, Sweden*

*Correspondence to: John F. Allen, Plant Biochemistry, Center for Chemistry and Chemical Engineering, Lund University, Box 124, SE-221 00 Lund, Sweden. E-mail: john.allen@plantbio.lu.se*

Received: 17 September 2002
Accepted: 25 November 2002

### Abstract

**Chloroplasts and mitochondria originated as bacterial symbionts. The larger, host cells acquired genetic information from their prokaryotic guests by lateral gene transfer. The prokaryotically-derived genes of the eukaryotic cell nucleus now function to encode the great majority of chloroplast and mitochondrial proteins, as well as many proteins of the nucleus and cytosol. Genes are copied and moved between cellular compartments with relative ease, and there is no established obstacle to successful import of any protein precursor from the cytosol. Yet chloroplasts and mitochondria have not abdicated all genes and gene expression to the nucleus and to cytosolic translation. What, then, do chloroplast- and mitochondrially-encoded proteins have in common that confers a selective advantage on the cytoplasmic location of their genes? The proposal advanced here is that co-location of chloroplast and mitochondrial genes with their gene products is required for rapid and direct regulatory coupling. Redox control of gene expression is suggested as the common feature of those chloroplast and mitochondrial proteins that are encoded *in situ*. Recent evidence is consistent with this hypothesis, and its underlying assumptions and predictions are described. Copyright © 2003 John Wiley & Sons, Ltd.**

**Keywords: cytoplasmic organelles; photosynthesis; respiration; gene expression; electron transport; cell evolution; redox regulation; CORR**

### The problem

Alberts *et al.* [2] describe the problem posed by the persistence of cytoplasmic genetic systems, as follows:

> Why do mitochondria and chloroplasts require their own separate genetic systems when other organelles that share the same cytoplasm, such as peroxisomes and lysosomes, do not? .... The reason for such a costly arrangement is not clear, and the hope that the nucleotide sequences of mitochondrial and chloroplast genomes would provide the answer has proved unfounded. We cannot think of compelling reasons why the proteins made in mitochondria and chloroplasts should be made there rather than in the cytosol.

### Hypotheses concerning the function of cytoplasmic genetic systems

### The lock-in hypothesis

According to the lock-in hypothesis [12], core components of multisubunit complexes must be synthesized, *de novo*, in the correct compartment. However, mechanisms of protein import and targeting [16,23,34,44] now effectively address the problem of specifying the correct locations for subunits of membrane protein complexes. Locking assembly into specific cellular compartments does not now seem likely as the primary function of organellar genomes.

### Transfer of genes from organelles to the nucleus is under way, but incomplete

This hypothesis is widely held [1,19,20,32]. A problem with the hypothesis is the non-random sample of genes that remain to be transferred [1,31]. The nuclear genome of *Arabidopsis thaliana* has a single insertion corresponding, with about 99% sequence similarity, to the whole mitochondrial genome [46], which suggests that gene

Copyright © 2003 John Wiley & Sons, Ltd.

transfer occurs with relative ease [21,42]. In addition, randomly-generated polypeptides are mitochondrially imported at high frequency [26].

### The frozen accident

'The frozen accident hypothesis' states that the evolutionary process of gene transfer from organelle to nucleus was under way when something happened that stopped it. One example [50] suggests that successful import into mitochondria of the precursor proteins arising from newly translocated genes proceeded for long enough, after the original endosymbiosis, for most of the symbiont-derived genes to be lost to the cell nucleus. The 'accident' that halted the process was the evolutionary origin of exocytosis and protein secretion. The 'frozen accident' seems incompatible with the precision and specificity of protein targeting [16,23,34,44].

### Hydrophobicity

The hydrophobicity hypothesis is that intrinsic membrane proteins must be synthesized *de novo* within organelles [13,37,50]. This hypothesis has some very clear counter-examples in chloroplasts. One is the large subunit of the enzyme ribulose-1,5-bisphosphate carboxylase-oxygenase (RubisCO), an abundant but water-soluble protein. RubisCO has a chloroplast-encoded large subunit and a nuclear-encoded small subunit [18], and serves as a model for coordination of nuclear and chloroplast gene expression. Other counter-examples from chloroplasts are the nuclear-encoded but hydrophobic subunits of the chloroplast light-harvesting complexes, LHC II and LHC I. Hydrophobicity is, in many cases, a feature of proteins that are encoded *in situ*, but does not seem to be, in itself, the issue.

### Some proteins cannot be imported

Protein import mechanisms appear mostly to rely on specific molecular chaperones for guided unfolding, prior to membrane insertion, and refolding, following translocation of the polypeptide into its destined compartment [18,22,24]. Since polypeptides are mostly 'threaded' through membranes, no special constraint on transport should be expected to arise from three-dimensional structure or surface properties of a protein. Holoproteins containing co-factors can be transported along with the apoprotein by the twin-arginine translocation (Tat) system [11,40,43]. The presence of a vesicular transport system in chloroplasts [51] also counts against the unimportability hypothesis. If vesicular transport into chloroplasts is possible it is difficult to see why there is any protein that is universally forbidden from changing compartments within the cell.

### Some genes cannot be moved

Mitochondria show departures from the otherwise universal genetic code [2,10]. There is, therefore, an obstacle to correct cytosolic translation of mRNA that is transcribed from unchanged nuclear copies of mitochondrial genes. Distinct mitochondrial 'dialects' of the genetic code must present a barrier to expression of nuclear copies of mitochondrial protein-coding genes. However, differences in the genetic code are unlikely to be the primary reason why genes are still present in mitochondria, since it is not clear why the code has to be different for some genes and not for others — the problem remains of why there are mitochondrial genes at all. The significance of this heterogeneity in coding is most unlikely to rest in the early origin of mitochondria, since the 'universal' code is employed by bacteria. Furthermore, the diversity of departures from the 'universal' code amongst mitochondria of different eukaryotic lineages suggests that these departures had independent origins.

### Co-location of genes and gene products for redox regulation of gene expression

The hypothesis of co-location for redox regulation of gene expression, here termed CORR, was systematically proposed in two articles [4,5], developed [9] and has been independently reviewed [38]. A more detailed, recent analysis is presented elsewhere [7]. Figure 1 illustrates the general idea of redox regulatory control giving rise to retention of genes in organelles. The CORR hypothesis is based on ten assumptions, or principles, as listed below.

1. *Bioenergetic organelles evolved from free-living bacteria.* The endosymbiont hypothesis

Copyright © 2003 John Wiley & Sons, Ltd.

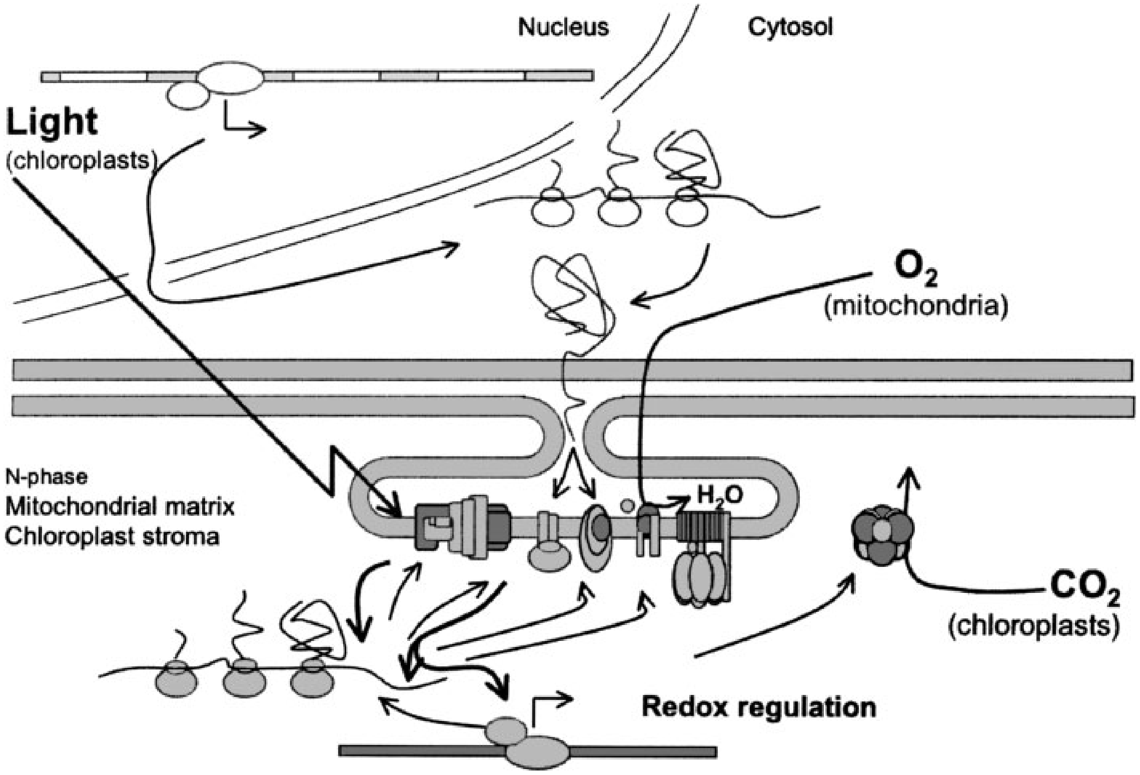

**Figure 1.** Gene expression and principal pathways of biosynthesis of subunits of protein complexes involved in photosynthesis in chloroplasts and oxidative phosphorylation in mitochondria. Dark DNA, RNA and protein subunits are located and synthesized in the mitochondrial matrix or chloroplast stroma; lighter protein subunits have genes (also lighter) in the nucleus, and are imported from the cytosol as precursors. White genes and ribosomal and protein subunits are nuclear–cytoplasmic and may be of archaebacterial origin. Dark and lighter genes and ribosomal and protein subunits are of bacterial origin. The major, variable environmental inputs are light and carbon dioxide for chloroplasts; oxygen for mitochondria. The CORR hypothesis assumes that it is beyond the ability of the nuclear–cytoplasmic system to respond rapidly and directly to changes in light, carbon dioxide and oxygen concentration. Such responses require continued redox regulation of gene expression. This regulation has therefore been retained from the eubacterial endosymbionts that were ancestral to chloroplasts and mitochondria, and its continued operation requires co-location of the genes concerned, with their gene products, within bioenergetic organelles was once controversial [27] but is now widely accepted [28,30].

2. *Gene transfer between the symbiont, or organelle, and nucleus may occur in either direction and is not selective for particular genes.* Nothing certain seems to be known about the mechanism of intra-cellular gene relocation [21,29,31,32], so there is no basis for asserting a mechanistic argument for selectivity. Gene transfer between organelle and nucleus seems to be a special case of lateral, or horizontal, gene transfer [14].
3. *There is no barrier to the successful import of any precursor protein, nor to its processing and assembly into a functional, mature form.* This principle forbids the existence of a protein, even a synthetic one, that cannot be imported by some means, as a precursor.
4. *Direct redox control of expression of certain genes was present in the bacterial progenitors of chloroplasts and mitochondria, and was vital for selectively advantageous cell function before, during and after the transition from bacterium to organelle. The mechanisms of this control have been conserved.* Experiments carried out *in vitro* on the products of protein synthesis [3,17] or RNA synthesis [25,33,53] are consistent with a general phenomenon of redox dependency of gene expression in bioenergetic organelles. Redox

Copyright © 2003 John Wiley & Sons, Ltd. 

control of chloroplast transcription has been demonstrated [8,35,36,48], and was prompted by this principle.

5. *For each gene under redox control, it is selectively advantageous for that gene to be retained and expressed only within the organelle.* The twin selective advantages of redox control are likely to be energetic efficiency and the suppression of the harmful side effects of electron transport operating on the wrong substrates.
6. *For each bacterial gene that survives and is not under redox control, it is selectively advantageous for that gene to be located in the nucleus and expressed only in the nucleus and cytosol. If the mature gene product functions in chloroplasts or mitochondria, the gene is first expressed in the form of a precursor for import.* There are several possible reasons for the selective advantage of nuclear location of genes for organellar proteins. One is the decreased probability of mutation arising from free-radical by-products of 'incorrect' electron transport [9,39]. Another is that organellar genes do not undergo recombination and are present in relatively small copy numbers, so that disadvantageous mutations will spread relatively quickly through a clonal population of organelles [41].
7. *For any species, the distribution of genes between organelle and nucleus is the result of selective forces that continue to operate.* Mitochondria that lose their function in aerobic respiration also lose their genomes, as seen in the relict mitochondria of microsporidia [15,49] and the 'mitosome' of *Entamoeba histolytica* [47]. In chloroplasts, loss of photosynthesis results in loss of photosynthetic genes. The examples of *Epifagus* (a parasitic higher plant) and the residual apicomplexan plastids of *Plasmodium* [52] and *Toxoplasma*, where some plastidic genetic system is retained, mean that some role for their gene products must be found which requires redox regulation of gene expression, if this principle is correct.
8. *Those genes for which redox control is always vital to cell function have gene products involved in, or closely connected with, primary electron transfer. These genes are always contained within the organelle.* This principle would be violated if any photosynthetic reaction centre core subunit or respiratory chain subunit currently organelle-encoded is found which functions in primary, vectorial electron transport and is nevertheless encoded in the nucleus and synthesized cytosolically as a precursor for import, all without any selective cost to the individual.
9. *Genes whose products contribute to the organelle genetic system itself, or whose products are associated with secondary events in energy transduction, may be contained in the organelle in one group of organisms but not in another, depending on the physiology and biochemistry of photosynthesis and respiration in the species concerned.* Even phylogenetically moderately-related species (within kingdoms) show differences in the location of some genes, e.g. the ATP synthase subunits of *Neurospora* and *Saccharomyces* [10].
10. *Components of the redox-signalling pathways upon which co-location for redox regulation depends are themselves not involved in primary electron transfer and so their genes have been relocated to the nucleus.* This principle states that the redox signalling pathways that are required for CORR should not themselves be expected to utilize components whose biosynthesis must be under direct redox control. Such components should therefore fall into the major category of organellar proteins and be imported as precursors from the cytosol.

### Conclusion and prospects

The CORR hypothesis is that the function of chloroplast and mitochondrial genomes is to provide co-location (of gene and gene product) for (evolutionary) continuity of redox regulation of gene expression. This hypothesis seems to be consistent with available evidence and applies equally to chloroplasts and mitochondria.

CORR predicts regulatory properties of known components of chloroplasts and mitochondria that suggest flexibility in energy metabolism. These components include the RubisCO large subunit and $CF_0$ and $F_0$ subunits of coupling ATPase in both chloroplasts and mitochondria. Redox regulation of the relative stoichiometry of components, as seen in regulation of chloroplast photosystem stoichiometry [35], may extend to $(C)F_0$-$(C)F_1$-ATPase, suggesting that the function of retention of genes for

Copyright © 2003 John Wiley & Sons, Ltd. 

one or more of these components is flexibility in the stoichiometries of $H^+$ to $e^-$ and $H^+$ to ATP in chemiosmotic coupling [6,45].

The central proposal of CORR is that chloroplasts and mitochondria contain genes for proteins whose function in electron transfer demands rapid, direct and unconditional redox regulatory control of their biosynthesis. This testable hypothesis makes many predictions, and may explain the otherwise puzzling distribution of genes between the nucleus and the cytoplasm of eukaryotic cells.

### Acknowledgements

I thank Carol Allen, William Martin and John Raven for discussion and correspondence. Research Grants from the Swedish Natural Sciences Research Council (NFR) and the Crafoord Foundation are gratefully acknowledged.

### References

1. Adams KL, Daley DO, Qiu YL, Whelan J, Palmer JD. 2000. Repeated, recent and diverse transfers of a mitochondrial gene to the nucleus in flowering plants. *Nature* **408**: 354–357.
2. Alberts B, Bray D, Lewis J, *et al*. 1994. *The Molecular Biology of the Cell*. Garland: New York.
3. Allen CA, Hakansson G, Allen JF. 1995. Redox conditions specify the proteins synthesized by isolated chloroplasts and mitochondria. *Redox Rep* **1**: 119–123.
4. Allen JF. 1993a. Control of gene-expression by redox potential and the requirement for chloroplast and mitochondrial genomes. *J Theor Biol* **165**: 609–631.
5. Allen JF. 1993b. Redox control of gene-expression and the function of chloroplast genomes — an hypothesis. *Photosynth Res* **36**: 95–102.
6. Allen JF. 2002. Photosynthesis of ATP-electrons, proton pumps, rotors, and poise. *Cell* **110**: 273–276.
7. Allen JF. 2003. The function of genomes in bioenergetic organelles. *Phil Trans R Soc Lond B Biol Sci* (in press).
8. Allen JF, Pfannschmidt T. 2000. Balancing the two photosystems: photosynthetic electron transfer governs transcription of reaction centre genes in chloroplasts. *Phil Trans R Soc Lond B Biol Sci* **355**: 1351–1357.
9. Allen JF, Raven JA. 1996. Free-radical-induced mutation vs. redox regulation: costs and benefits of genes in organelles. *J Mol Evol* **42**: 482–492.
10. Attardi G, Schatz G. 1988. Biogenesis of mitochondria. *Ann Rev Cell Biol* **4**: 289–333.
11. Berks BC, Sargent F, Palmer T. 2000. The Tat protein export pathway. *Mol Microbiol* **35**: 260–274.
12. Bogorad L. 1975. Evolution of organelles and eukaryotic genomes. *Science* **188**: 891–898.
13. Claros MG, Perea J, Shu Y, *et al*. 1995. Limitations to *in vivo* import of hydrophobic proteins into yeast mitochondria. The case of a cytoplasmically synthesized apocytochrome b. *Eur J Biochem* **228**: 762–771.
14. Doolittle WF. 1999. Phylogenetic classification and the universal tree. *Science* **284**: 2124–2129.
15. Embley TM, Martin W. 1998. Molecular evolution — a hydrogen-producing mitochondrion. *Nature* **396**: 517–519.
16. Gabriel K, Buchanan SK, Lithgow T. 2001. The alpha and the beta: protein translocation across mitochondrial and plastid outer membranes. *Trends Biochem Sci* **26**: 36–40.
17. Galvis MLE, Allen JF, Hakansson G. 1998. Protein synthesis by isolated pea mitochondria is dependent on the activity of respiratory complex II. *Curr Genet* **33**: 320–329.
18. Gatenby AA, Ellis RJ. 1990. Chaperone function — the assembly of ribulose bisphosphate carboxylase-oxygenase. *Ann Rev Cell Biol* **6**: 125–149.
19. Gray MW. 2000. Evolutionary biology — mitochondrial genes on the move. *Nature* **408**: 302–305.
20. Gray MW, Burger G, Lang BF. 1999. Mitochondrial evolution. *Science* **283**: 1476–1481.
21. Henze K, Martin W. 2001. How do mitochondrial genes get into the nucleus? *Trends Genet* **17**: 383–387.
22. Jackson-Constan D, Akita M, Keegstra K. 2001. Molecular chaperones involved in chloroplast protein import. *Biochim Biophys Acta Mol Cell Res* **1541**: 102–113.
23. Koehler CM, Merchant S, Schatz G. 1999. How membrane proteins travel across the mitochondrial intermembrane space. *Trends Biochem Sci* **24**: 428–432.
24. Komiya T, Rospert S, Schatz G, Mihara K. 1997. Binding of mitochondrial precursor proteins to the cytoplasmic domains of the import receptors Tom70 and Tom20 is determined by cytoplasmic chaperones. *FASEB J* **11**: 1196.
25. Konstantinov YM, Lutsenko GN, Podsosonny VA. 1995. Genetic functions of isolated maize mitochondria under model changes of redox conditions. *Biochem Mol Biol Int* **36**: 319–326.
26. Lemire BD, Fankhauser C, Baker A, Schatz G. 1989. The mitochondrial targeting function of randomly generated peptide sequences correlates with predicted helical amphiphilicity. *J Biol Chem* **264**: 20 206–20 215.
27. Mahler HR, Raff RA. 1975. The evolutionary origin of the mitochondrion: a non-symbiotic model. *Int Rev Cytol* **43**: 1–124.
28. Martin W. 1999. A briefly argued case that mitochondria and plastids are descendants of endosymbionts, but that the nuclear compartment is not. *Proc R Soc Lond B Biol Sci* **266**: 1387–1395.
29. Martin W, Herrmann RG. 1998. Gene transfer from organelles to the nucleus: how much, what happens, and why? *Plant Physiol* **118**: 9–17.
30. Martin W, Hoffmeister M, Rotte C, Henze K. 2001. An overview of endosymbiotic models for the origins of eukaryotes, their ATP-producing organelles (mitochondria and hydrogenosomes), and their heterotrophic lifestyle. *Biol Chem* **382**: 1521–1539.
31. Martin W, Stoebe B, Goremykin V, *et al*. 1998. Gene transfer to the nucleus and the evolution of chloroplasts. *Nature* **393**: 162–165.
32. Palmer JD. 1997. Organelle genomes: going, going, gone! *Science* **275**: 790–791.
33. Pearson CK, Wilson SB, Schaffer R, Ross AW. 1993. NAD turnover and utilization of metabolites for RNA-synthesis

Copyright © 2003 John Wiley & Sons, Ltd. 

in a reaction sensing the redox state of the cytochrome-B(6)F complex in isolated-chloroplasts. *Eur J Biochem* **218**: 397–404.
34. Pfanner N, Wiedemann N. 2002. Mitochondrial protein import: two membranes, three translocases. *Curr Opin Cell Biol* **14**: 400–411.
35. Pfannschmidt T, Nilsson A, Allen JF. 1999a. Photosynthetic control of chloroplast gene expression. *Nature* **397**: 625–628.
36. Pfannschmidt T, Nilsson A, Tullberg A, Link G, Allen JF. 1999b. Direct transcriptional control of the chloroplast genes psbA and psaAB adjusts photosynthesis to light energy distribution in plants. *IUBMB Life* **48**: 271–276.
37. Popot JL, de Vitry C. 1990. On the microassembly of integral membrane proteins. *Ann Rev Biophys Biophys Chem* **19**: 369–403.
38. Race HL, Herrmann RG, Martin W. 1999. Why have organelles retained genomes? *Trends Genet* **15**: 364–370.
39. Raven JA, Johnston AM, Parsons R, Kubler J. 1994. The influence of natural and experimental high $O_2$ concentrations on $O_2$-evolving phototrophs. *Biol Rev Camb Phil Soc* **69**: 61–94.
40. Robinson C, Bolhuis A. 2001. Protein targeting by the twin-arginine translocation pathway. *Nat Rev Mol Cell Biol* **2**: 350–356.
41. Saccone C, Gissi C, Lanave C, *et al*. 2000. Evolution of the mitochondrial genetic system: an overview. *Gene* **261**: 153–159.
42. Salzberg SL, White O, Peterson J, Eisen JA. 2001. Microbial genes in the human genome: lateral transfer or gene loss? *Science* **292**: 1903–1906.
43. Sargent F, Berks BC, Palmer T. 2002. Assembly of membrane-bound respiratory complexes by the Tat protein-transport system. *Arch Microbiol* **178**: 77–84.
44. Schatz G. 1998. Protein transport — the doors to organelles. *Nature* **395**: 439–440.
45. Schemidt RA, Qu J, Williams JR, Brusilow WS. 1998. Effects of carbon source on expression of $F_0$ genes and on the stoichiometry of the c subunit in the F1F0 ATPase of *Escherichia coli*. *J Bacteriol* **180**: 3205–3208.
46. Stupar RM, Lilly JW, Town CD, *et al*. 2001. Complex mtDNA constitutes an approximate 620 kb insertion on *Arabidopsis thaliana* chromosome 2: implication of potential sequencing errors caused by large-unit repeats. *Proc Natl Acad Sci USA* **98**(9): 5099–5103.
47. Tovar J, Fischer A, Clark CG. 1999. The mitosome, a novel organelle related to mitochondria in the amitochondrial parasite *Entamoeba histolytica*. *Mol Microbiol* **32**: 1013–1021.
48. Tullberg A, Alexciev K, Pfannschmidt T, Allen JF. 2000. Photosynthetic electron flow regulates transcription of the psaB gene in pea (*Pisum sativum* L.) chloroplasts through the redox state of the plastoquinone pool. *Plant Cell Physiol* **41**: 1045–1054.
49. van der Giezen M, Slotboom DJ, Horner DS, *et al*. 2002. Conserved properties of hydrogenosomal and mitochondrial ADP/ATP carriers: a common origin for both organelles. *EMBO J* **21**: 572–579.
50. Von Heijne G. 1986. Why mitochondria need a genome. *FEBS Lett* **198**: 1–4.
51. Westphal S, Soll J, Vothknecht UC. 2001. A vesicle transport system inside chloroplasts. *FEBS Lett* **506**: 257–261.
52. Wilson RJ, Denny PW, Preiser PR, *et al*. 1996a. Complete gene map of the plastid-like DNA of the malaria parasite *Plasmodium falciparum*. *J Mol Biol* **261**: 155–172.
53. Wilson SB, Davidson GS, Thomson LM, Pearson CK. 1996b. Redox control of RNA synthesis in potato mitochondria. *Eur J Biochem* **242**: 81–85.

Copyright © 2003 John Wiley & Sons, Ltd. 
